# Simple Approach to Synthesize Amino-Functionalized Carbon Dots by Carbonization of Chitosan

**DOI:** 10.1038/srep31100

**Published:** 2016-08-05

**Authors:** Xin Liu, Jinhui Pang, Feng Xu, Xueming Zhang

**Affiliations:** 1 Beijing Key Laboratory of Lignocellulosic Chemistry, Beijing Forestry University, Beijing 100083, P. R. China

## Abstract

Carbon dots (CDs) as a new series of fluorescent nanomaterials have drawn great attention in recent years owning to their unique properties. In this paper, a simple carbonization approach to synthesize amino-functionalized CDs was developed by using chitosan as the carbon precursor. The as-prepared CDs possessed desirable amino function group on their surface and exhibited bright luminescence with absolute quantum yield (QY) of 4.34%, excitation-, pH-dependent and up-conversion fluorescence behaviors. Furthermore, we have investigated the cytotoxicity and biocompatibility of the as-prepared CDs, which demonstrated that the as-prepared CDs have the potential applications in biosensing, cellular imaging and drug delivery.

Carbon dots (CDs) as a new member of carbon nanomaterials have drawn a great deal of attention, owning to their chemical stability, excellent water solubility, tuneable fluorescence properties, low cost, low toxicity, good biocompatibility and environmental friendliness^1^,^2^,^3^,^4^. These outstanding merits make them show promising prospects as benign candidates for various potential applications, such as fluorescent markers, bioimaging agents, photocatalysts, sensors and optoelectronic devices^5^,^6^,^7^,^8^,^9^. Therefore, exploring alternative routes and starting materials for the synthesis of CDs are highly needed.

Up to now, a variety of methods have been developed to synthesize CDs, which can be roughly classified into “top-down” and “bottom-up” approaches^10^. The “top-down” approach includes chemical oxidation, electrochemical synthesis, arc-discharge and laser-ablation, where the CDs are formed from larger carbon materials^11^. The “bottom-up” approach refers to obtaining CDs from molecular precursors. Generally, “bottom-up” approach consists of microwave/ultrasonic preparation, plasma treatment, hydrothermal/acidic oxidation route and carbonizing organics route^12^,^13^,^14^. Nevertheless, many of these synthesis methods involve expensive or toxic starting materials, high temperature, long reaction time and further surface-passivation^15^,^16^.

In recent years, the preparation of fluorescent CDs without using organic chemicals in a simple, economical and environmentally friendly way is of great interest, which is so-called the green chemistry concept^17^. Some green synthetic routes have been developed for the preparation of CDs by using inexpensive and renewable resources as starting materials, like watermelon peels, orange juice, chicken eggs, gelatin, etc^11^,^18^,^19^. Remarkably, chitosan is the *N*-deacetylated derivative of chitin (a naturally abundant mucopolysaccharide), containing high amount of amino (-NH_2_) and hydroxyl (-OH) functional groups, also accepted as carbon source to synthesize CDs owing to its natural, nontoxic and biocompatible properties^20^,^21^.

In this work, we presented a very simple approach to synthesize amino-functionalized CDs by one-step carbonization treatment of chitosan (Fig. 1), which was scarcely studied before. In this method, neither a strong acid solvent nor surface passivation reagent was used. Both the formation and the functionalization of CDs were accomplished simultaneously. The as-prepared amino-functionalized CDs exhibited excellent fluorescence together with excitation-, pH-dependent and up-conversion fluorescence behaviors. Besides, due to their low toxicity and fine aqueous dispersibility, the as-prepared CDs showed great potential in biosensing, cellular imaging and drug delivery applications.

## Results and Discussion

### Physical structure of as-prepared CDs

**Figure 2:**
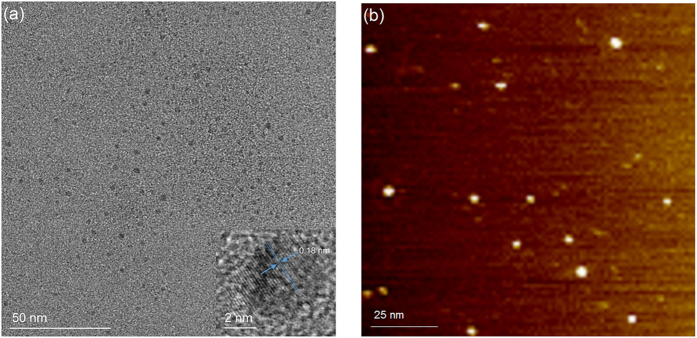
TEM (**a**) and AFM (**b**) images of as-prepared CDs.

**Figure 3:**
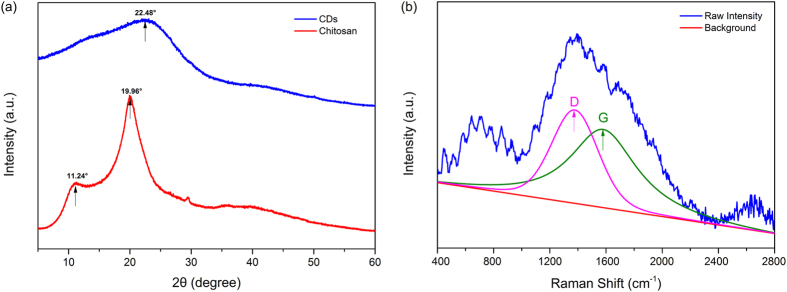
(**a**) XRD patterns of chitosan and as-prepared CDs and (**b**) Raman spectrum of as-prepared CDs.

The transmission electron microscopy (TEM) and atomic-force microscopy (AFM) images (Fig. 2) illustrated that the as-prepared CDs were quasi-spherical and monodisperse with size distribution between 1–6 nm in diameter. Besides, the high resolution transmission electron microscopy (HRTEM) image (inset of Fig. 2(a)) clearly showed the lattice spacing of 0.18 nm, which might be assigned to the (102) facet of graphitic carbon^15^. The X-ray diffraction (XRD) pattern of chitosan and as-prepared CDs were displayed in Fig. 3(a). The spectrum of chitosan showed distinct diffraction peaks at 11.24 and 19.96°, corresponding to the amorphous peak and crystalline peak, separately^22^. After carbonization, the crystallinity of chitosan diminished and the spectrum of as-prepared CDs showed a broad peak at 2θ = 22.48°, similar to the graphite lattice spacing, which was attributed to highly disordered carbon atoms^23^,^24^. Additionally, the Raman spectrum of as-prepared CDs (Fig. 3(b)) revealed that although the fluorescence background signal was very strong, a relatively strong D band at 1378 cm^−1^ and G band at 1580 cm^−1^ could be obviously observed, and the ratio of intensities of I_D_/I_G_ was calculated to be 1.15, indicating the presence of more defects or amorphous carbon as compared with sp^2^ hybridization in as-prepared CDs^25^,^26^,^27^, which coincided with the HRTEM and XRD results.

### Chemical structure of as-prepared CDs

**Figure 4:**
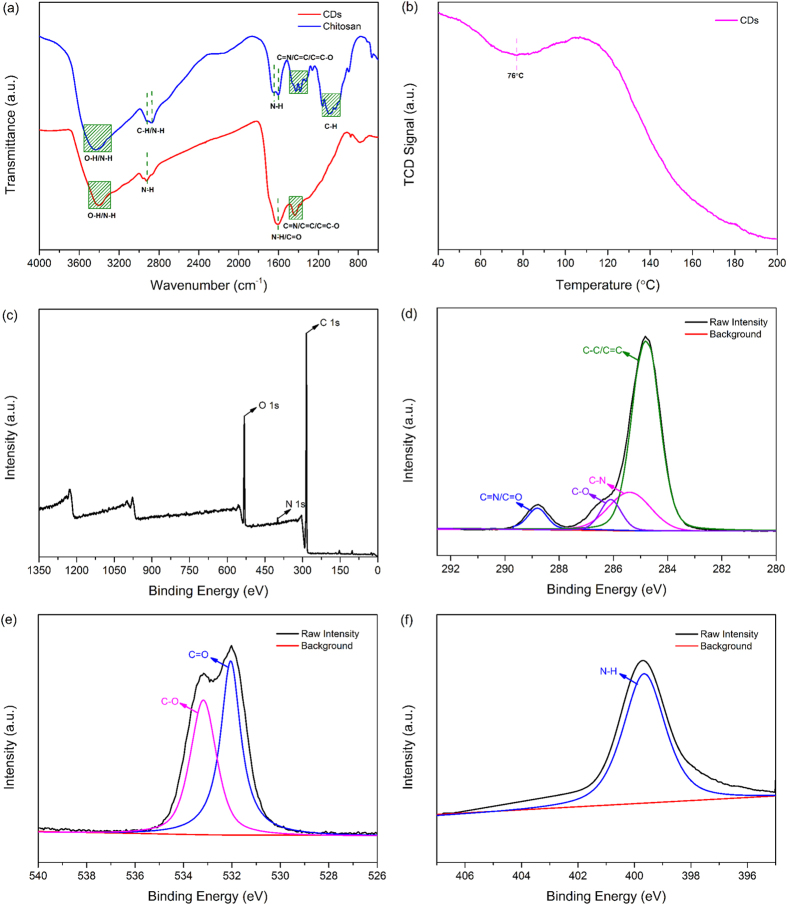
(**a**) FT-IR spectra of chitosan and as-prepared CDs, (**b**)CO_2_-TPD profile, (**c**) Whole survey XPS spectrum, (**d**) C 1s XPS spectrum, (**e**) O 1s XPS spectrum and (**f**) N 1s XPS spectrum of as-prepared CDs.

The Fourier transform infrared (FT-IR) spectra of chitosan and as-prepared CDs were presented in Fig. 4(a). The basic characteristic peaks of chitosan appeared at 3438 cm^−1^ (O-H and N–H stretching vibrations), 2920 and 2875 cm^−1^ (C-H stretching vibration), 1643 and 1602 cm^−1^ (N-H bending vibration), 1493–1245 cm^−1^ (C=C, C=N and C=C–O groups) and 1165–992 cm^−1^ (C-H bending vibrations of the pyranose ring)^14^. However, for the as-prepared CDs, the adsorption of O-H and N-H stretching vibration at 3401 cm^−1^ were decreased. Meanwhile, the C-H bending vibrations associated with pyranose ring in chitosan were completely disappeared. These differences between chitosan and as-prepared CDs might be ascribed to the decomposition of the chitosan chain and pyranose ring during the carbonization process. Notably, the bands at 2930 and 1615 cm^−1^ corresponded to N-H bending vibration of amino group increased^28^. The temperature programmed desorption of CO_2_ (CO_2_-TPD) profile of as-prepared CDs (Fig. 4(b)) suggested a distinct desorption of CO_2_ centered around 76 °C, similar to previous reports for amino-modified solids^29^, which might be due to the interaction between amino-functionalized CDs and CO_2_ via a chemisorption mechanism. The general formula of interaction between the functional amine and the CO_2_ molecules results in the formation of ammonium carbamates under anhydrous conditions can be seen in Equation (1) 30,31:


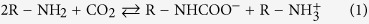


Also, the zeta-potential of as-prepared CDs was positive when the pH was below 6.67, indicating the existence of amine groups on the surface of as-prepared CDs together with the FT-IR and CO_2_-TPD results^32^.

X-ray photoelectron spectroscopy (XPS) measurement was carried out to further investigate functional groups presented on the surface of as-prepared CDs. The whole XPS survey spectrum of the as-prepared CDs (Fig. 4(c)) exhibited three obvious peaks at 284.81, 399.33, and 532.3 eV, which were attributed to C 1s, N 1s and O 1s, respectively. The C 1s XPS spectrum (Fig. 4(d)) can be further deconvoluted into four carbon states at 284.82, 285.40, 286.12 and 288.79 eV, which were attributed to C-C/C=C, C–N, C–O and C=N/C=O respectively and might result in a series of emissive traps between π and π* states of C=C^15^. The O 1s spectrum (Fig. 4(e)) showed two peaks at 532.01 and 533.17 eV, which were assigned to the C=O and C-O bands, respectively^28^,^33^. Furthermore, the N 1s spectrum (Fig. 4(f)) revealed an apparent peak at 399.83 eV, which was associated with N-H band^14^. The results from XPS spectra showed that the presence of hydrophilic functional groups on the surface of the as-prepared CDs without further modification. And these groups made as-prepared CDs aqueous dispersible and made a significant contribution to their optical properties^34^.

### Optical properties of as-prepared CDs

**Figure 5:**
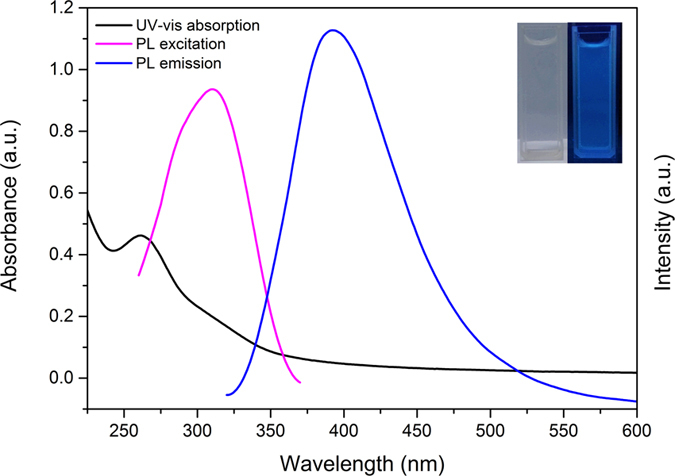
UV-Vis absorption, PL excitation and PL emission spectra of as-prepared CDs solution. Inset are the photos of as-prepared CDs solution under sunlight (left) and 365 nm UV lamp (right) illumination.

Although the fluorescence emission mechanisms remain debatable, two classes of fluorescence emission mechanisms have been proposed. The first class mechanism is related to the bandgap transitions caused by conjugated π-domains, while the second class mechanism arises from the existence of multiple surface defects^35^. The Ultraviolet-visible (UV-Vis) absorption and photoluminescence (PL) spectra of as-prepared CDs were investigated as shown in Fig. 5. From UV-Vis absorption, it was obviously that a strong UV-Vis absorption peak located at 261 nm, which typically assigned to the π-π* transition of aromatic sp^2^ domains from the carbon core^14^,^36^. As shown in the PL spectra, when excited at the maximum excitation wavelength of 310 nm, the as-prepared CDs exhibited a strong PL peak at 390 nm. The aqueous solution of as-prepared CDs was nearly transparent under visible light, while it displayed strong blue fluorescence under UV light (365 nm) excitation. (inset, Fig. 5)

**Figure 6:**
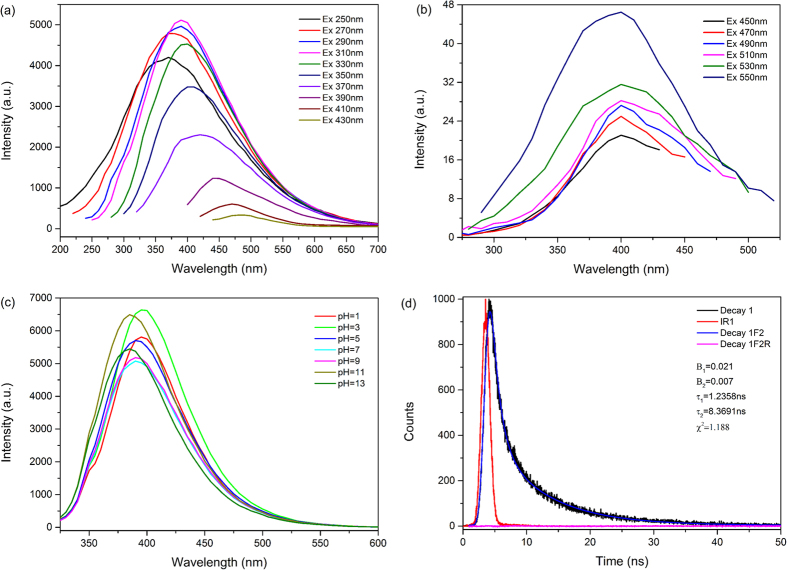
(**a**) Excitation-dependent PL emission spectra and (**b**) Up-conversion PL emission spectra of as-prepared CDs. (**c**) PL spectra of as-prepared CDs dispersed in different pH solutions and (**d**) Time-resolved PL decay and fitting curves for as-prepared CDs.

Furthermore, the phenomenon of excitation dependent was clearly observed in Fig. 6(a). The emission spectra were sensitive to the excitation wavelength and the emission peak varied from 370 nm to 490 nm as the excitation wavelength increased from 250 nm to 430 nm. When the excitation wavelength increased from 250 to 310 nm, the emission peak become stronger and redshift. When excited from 310 to 430 nm, the emission peak kept redshift with remarkably decreased intensities. The excitation-dependent and redshift optical properties were similar to previous studies^37^,^38^,^39^. As shown in Fig. 6(b), the up-conversion fluorescence emission phenomenon was observed that when as-prepared CDs were excited by longer wavelength (450–550 nm), shorter wavelength (400 nm) was emitted, which may be caused by the two or multiphoton active process^40^. Figure 6(c) illustrated that the optical responses of the as-prepared CDs with pH values ranging from 1 to 13. It was clearly that PL emission wavelength of as-prepared CDs were stable in the pH range of 5 to 9. In strong acidic condition, the emission wavelength evolved to the longer wavelength, while moved to shorter wavelength in strong basic solution, implying that the as-prepared CDs were very sensitive to acid/base solvent. These tunable fluorescence emission properties of as-prepared CDs were particular attractive for sensing, *in vivo* bioimaging and synthesizing novel photocatalysts^7^,^11^,^40^.

Fluorescence decay curve and the exponential fitting curve of as-prepared CDs were showed in Fig. 6(d). The fitting formula is:


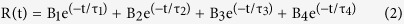


where t is time, B_1_, B_2_, B_3_and B_4_ are fractional intensities, τ_1_, τ_2_, τ_3_ and τ_4_ are fluorescence lifetime. The parameters generated from iterative reconvolution of the decay with the instrument response function (IRF) were listed in the inset of Fig. 6(d). And according to formula:


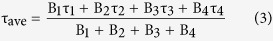


we calculated the amplitude-weighted average fluorescence lifetime (τ_ave_) of as-prepared CDs to be 6.0921 ns, which suggested that the as-prepared CDs are suitable for optoelectronic and biological application^17^,^41^. Additionally, the value of absolute quantum yield (QY) was measured to be 4.34%, which was comparable to the reported carbon dots^2^,^42^,^43^.

### Cytotoxicity and cells imaging

**Figure 7:**
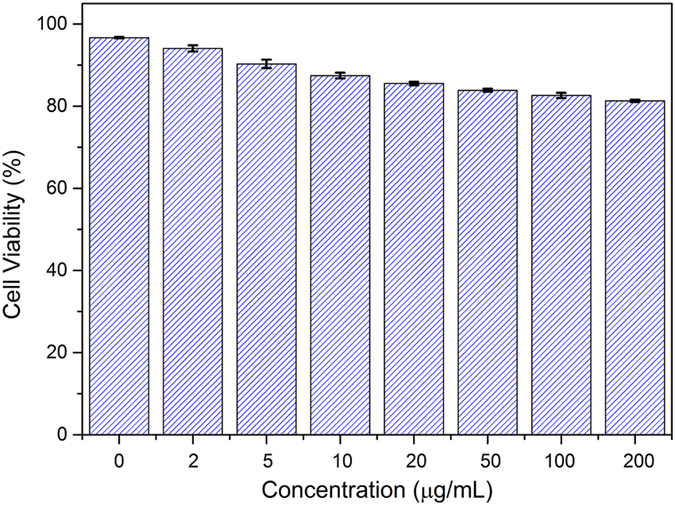
Cell viability of LLC cells in the presence of different concentration of as-prepared CDs.

**Figure 8:**
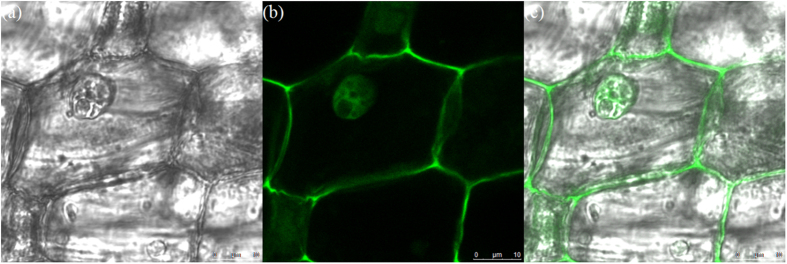
CLSM images of onion root tip cells labeled with as-prepared CDs at 25 °C for 12 h (**a**) Bright field, (**b**) Fluorescent image excited with a 405 nm laser and (**c**) Overlap of the corresponding bright field image and the fluorescence image.

CCK-8 assay was used to evaluate the cytotoxicity of the CDs to living cells. As shown in Fig. 7, the cell viability were estimated to be greater than 80% after 24 h of incubation upon addition of the as-prepared CDs over concentration ranged from 0 to 200 μg/mL, which confirmed the low toxicity, excellent biocompatibility and safety *in vitro* and *in vivo* applications of the as-prepared CDs^6^,^44^. Furthermore, in order to investigate the imaging capability, we induced the as-prepared CDs into onion root tip cells. The confocal laser scanning microscopy (CLSM) images of onion root tip cells labeled with the as-prepared CDs for 12 h at 25 °C were shown in Fig. 8. Obviously, the as-prepared CDs have penetrated into the cells and labeled both the cell nuclei and membrane and shown significant green fluorescence emission under the 405 nm wavelength excitation. This result indicated that the as-prepared CDs possessed good biocompatibility and could serve in bioimaging and drug delivery applications^45^,^46^.

## Conclusions

In summary, the amino-functionalized CDs have been successfully prepared by a simple carbonization approach. This green, cheap and convenient process represented a potential advance for large-scale production. The as-prepared CDs possessed desirable amino function group on the surface and exhibited bright luminescence with absolute quantum yield (QY) of 4.34%, excitation-, pH-dependent and up-conversion fluorescence behaviors. Furthermore, the as-prepared CDs showed good stability, less toxicity, biocompatibility and excellent fluorescence imaging ability. In the future, the as-prepared CDs could be utilized promisingly in optoelectronic materials, photocatalysts, cellular imaging and drug delivery applications.

## Methods

### Materials

All chemicals, including chitosan (Sinopharm Chemical Reagent Co., Ltd. China), sulfuric acid (H_2_SO_4_; Beijing Chemical Works, China), sodium hydroxide (NaOH; Beijing Chemical Works, China) and phosphate buffer saline (PBS; Leagene, China), were in analytical grade and used without further purification. Deionized water was used throughout the whole experiment.

### Preparation of CDs

CDs were prepared via carbonization of chitosan at 300 °C for 2 h at a heating rate of 5 °C/min under a nitrogen atmosphere. After cooled down to room temperature, the dark brown products were mechanically ground to fine powders. After that, obtained powders were dispersed in water, and the CDs were collected by filtrating for further characterization and use.

### Characterization

Transmission electron microscopy (TEM) and high resolution transmission electron microscopy (HRTEM) micrographs were recorded on JEM-2100F (JEOL, Japan) operating at 200 kV. Atomic-force microscopy (AFM) investigation was done by a scanning probe microscope (BRUKER MULTIMADE 8, Bruker, USA). The crystal structure of samples was analyzed by an X-ray diffractometer (SHIMADZU XRD-6000, Shimadzu corporation, Japan) with Cu radiation (λ = 0.154 nm) at 40 kV and 40 mA. The Raman spectrum was recorded on Perkin Elmer Spectrum GX (Perkin Elmer, UK) at room temperature. Fourier-transform infrared (FT-IR) spectroscopy data were acquired using a Perkin Elmer Spotlight 400 imaging system (Perkin Elmer, UK). The X-ray photoelectron spectroscopy (XPS) measurements were performed on Thermo Scientific Escalab 250Xi XPS system (Thermo Fisher Scientific Ltd., UK) using Al Kα source. The temperature programmed desorption of CO_2_ (CO_2_-TPD) measurement was carried out on an AutoChem II 2920 analyser (Micrometrics, USA) fitted with a thermal conductivity detector (TCD). Ultraviolet-visible (UV-Vis) absorption spectra were carried out with UV2310II spectrometer (Techcomp, China). The fluorescence spectra were measured with a fluorescence spectrometer (F-7000, Hitachi High Tech CO., Japan). Fluorescence decay curves and photoluminescence (PL) absolute quantum yield (QY) for as-prepared CDs were measured at room temperature using an Edinburgh FLS 980 spectrometer (Edinburgh Instruments, UK) equipped with an integrating sphere under 310 nm excitation from a 450 W xenon lamp. The absolute fluorescence quantum yield was calculated as follows:


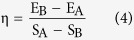


where η is the absolute fluorescence quantum yield, the indices “A” and “B” referred to the reference sample (deionized water) and carbon dots aqueous solution, separately, and S_A_, S_B_, E_A_ and E_B_ referred to the integral of the scans. The specific calculation was handled by using the Edinburgh FLS 980 software package (Quantum Yield Wizard)^47^,^48^.

### Effect of pH on the PL of as-prepared CDs

The as-prepared CDs aqueous solutions were adjusted to the various target pH values by adding diluted H_2_SO_4_ or NaOH solution, then the corresponding fluorescence spectra were measured upon excitation at 310 nm separately.

### Cytotoxicity evaluation

The cytotoxicity of as-prepared CDs was evaluated by a CCK-8 assay^44^. Lewis Lung Carcinoma (LLC) cells were seeded at a density of 4 × 10^3^ cells per well in 180 mL culture medium and incubated for 24 h. Then, the cells were treated with different concentrations of as-prepared CDs solution at 37 °C in a humidified incubator with 5% CO_2_ for 24 h. Then, 20 mL of CCK-8 solution was added to each well and incubated for 1 h at 37 °C. The absorbance at 450 nm was measured using an infinite M200 microplate spectrophotometer (Tecan, Switzerland). The cell viability was expressed as percentage of absorbance relative to control, and the control was obtained in the absence of as-prepared CDs.

### Cell imaging

Onion root tip cells were incubated with as-prepared CDs solution for 12 h at 25 °C, then washed with PBS for several times. The cellular localization was visualized using a confocal laser scanning microscope (SP-5, Leica, Germany) with laser excitation of 405 nm^49^,^50^.

## Additional Information

**How to cite this article**: Liu, X. *et al.* Simple Approach to Synthesize Amino-Functionalized Carbon Dots by Carbonization of Chitosan. *Sci. Rep.* **6**, 31100; doi: 10.1038/srep31100 (2016).

## Figures and Tables

**Figure 1:**
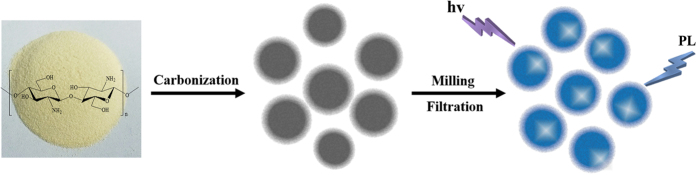
Schematic illustration of the preparation of CDs from chitosan.

## References

[b1] Zhu S. *et al.* Highly photoluminescent carbon dots for multicolor patterning, sensors, and bioimaging. Angew Chem Int Ed Engl. 52, 3953–3957 (2013). 23450679 10.1002/anie.201300519

[b2] Wang C., Sun D., Zhuo K., Zhang H. & Wang J. Simple and green synthesis of nitrogen-, sulfur-, and phosphorus-co-doped carbon dots with tunable luminescence properties and sensing application. RSC Adv. 4, 54060–54065 (2014).

[b3] Sun Y. P. *et al.* Host-Guest Carbon Dots for Enhanced Optical Properties and Beyond. Sci Rep. 5, 12354 (2015). 26196598 10.1038/srep12354 PMC4508828

[b4] Liu Y., Zhao Y. & Zhang Y. One-step green synthesized fluorescent carbon nanodots from bamboo leaves for copper(II) ion detection. Sensors Actua B-Chem. 196, 647–652 (2014).

[b5] Jamieson T. *et al.* Biological applications of quantum dots. Biomaterials. 28, 4717–4732 (2007). 17686516 10.1016/j.biomaterials.2007.07.014

[b6] Luo P. G. *et al.* Carbon “quantum” dots for optical bioimaging. J. Mater. Chem. B. 1, 2116–2127 (2013). 10.1039/c3tb00018d 32260843

[b7] Li H., Kang Z., Liu Y. & Lee S.-T. Carbon nanodots: synthesis, properties and applications. J. Mater. Chem. 22, 24230–24253 (2012).

[b8] Cao L. *et al.* Carbon dots for multiphoton bioimaging. J Am Chem Soc. 129, 11318–11319 (2007). 17722926 10.1021/ja073527l PMC2691414

[b9] Araújo T. C. *et al.* Hybrid heterostructures based on hematite and highly hydrophilic carbon dots with photocatalytic activity. Appl Catal B-Environ. 182, 204–212 (2016).

[b10] Wang Y. & Hu A. Carbon quantum dots: synthesis, properties and applications. J. Mater. Chem. C. 2, 6921–6939 (2014).

[b11] Lim S. Y., Shen W. & Gao Z. Carbon quantum dots and their applications. Chem Soc Rev. 44, 362–381 (2015). 25316556 10.1039/c4cs00269e

[b12] Liu S.-S. *et al.* Hair-derived carbon dots toward versatile multidimensional fluorescent materials. J. Mater. Chem. C. 2, 6477–6483 (2014).

[b13] Guo X., Wang C. F., Yu Z. Y., Chen L. & Chen S. Facile access to versatile fluorescent carbon dots toward light-emitting diodes. Chem. Commun. 48, 2692–2694 (2012). 10.1039/c2cc17769b 22306963

[b14] Liu Y. *et al.* One-step synthesis of robust nitrogen-doped carbon dots: acid-evoked fluorescence enhancement and their application in Fe3 + detection. J. Mater. Chem. A 3, 17747–17754 (2015).

[b15] Sahu S., Behera B., Maiti T. K. & Mohapatra S. Simple one-step synthesis of highly luminescent carbon dots from orange juice: application as excellent bio-imaging agents. Chem Commun (Camb). 48, 8835–8837 (2012). 22836910 10.1039/c2cc33796g

[b16] Yang Y. *et al.* One-step synthesis of amino-functionalized fluorescent carbon nanoparticles by hydrothermal carbonization of chitosan. Chem Commun (Camb). 48, 380–382 (2012). 22080285 10.1039/c1cc15678k

[b17] Xue M., Zou M., Zhao J., Zhan Z. & Zhao S. Green preparation of fluorescent carbon dots from lychee seeds and their application for the selective detection of methylene blue and imaging in living cells. J. Mater. Chem. B. 3, 6783–6789 (2015). 10.1039/c5tb01073j 32262471

[b18] Zhu L., Yin Y., Wang C.-F. & Chen S. Plant leaf-derived fluorescent carbon dots for sensing, patterning and coding. J. Mater. Chem. C. 1, 4925–4932 (2013).

[b19] Song Y., Zhu S. & Yang B. Bioimaging based on fluorescent carbon dots. RSC Advances. 4, 27184–27200 (2014).

[b20] Rinaudo M. Chitin and chitosan: Properties and applications. Prog. Polym. Sci. 31, 603–632 (2006).

[b21] Kumar M. N. V. R. A review of chitin and chitosan applications. React. Functi. Polym. 46, 1–27 (2000).

[b22] Zhang Y., Xue C., Xue Y., Gao R. & Zhang X. Determination of the degree of deacetylation of chitin and chitosan by X-ray powder diffraction. Carbohydr Res. 340, 1914–1917 (2005). 15963961 10.1016/j.carres.2005.05.005

[b23] Wang F., Kreiter M., He B., Pang S. & Liu C. Y. Synthesis of direct white-light emitting carbogenic quantum dots. Chem Commun (Camb). 46, 3309–3311 (2010). 20372692 10.1039/c002206c

[b24] Wang W., Li Y., Cheng L., Cao Z. & Liu W. Water-soluble and phosphorus-containing carbon dots with strong green fluorescence for cell labeling. J. Mater. Chem. B. 2, 46–48 (2014). 10.1039/c3tb21370f 32261297

[b25] Qu S., Wang X., Lu Q., Liu X. & Wang L. A biocompatible fluorescent ink based on water-soluble luminescent carbon nanodots. Angew Chem Int Ed Engl. 51, 12215–12218 (2012). 23109224 10.1002/anie.201206791

[b26] Machado C. E. *et al.* Influence of Inert and Oxidizing Atmospheres on the Physical and Optical Properties of Luminescent Carbon Dots Prepared through Pyrolysis of a Model Molecule. Chem. Eur. J. 22, 4556–4563 (2016). 26845751 10.1002/chem.201504234

[b27] Amama P. B., Cola B. A., Sands T. D., Xu X. & Fisher T. S. Dendrimer-assisted controlled growth of carbon nanotubes for enhanced thermal interface conductance. Nanotechnology. 18, 385303 (2007).

[b28] Liu S. *et al.* Hydrothermal treatment of grass: a low-cost, green route to nitrogen-doped, carbon-rich, photoluminescent polymer nanodots as an effective fluorescent sensing platform for label-free detection of Cu(II) ions. Adv Mater. 24, 2037–2041 (2012). 22419383 10.1002/adma.201200164

[b29] Madden D. & Curtin T. Carbon dioxide capture with amino-functionalised zeolite-β: A temperature programmed desorption study under dry and humid conditions. Microporous Mesoporous Mater. 228, 310–317 (2016).

[b30] Knowles G. P., Graham J. V., Delaney S. W. & Chaffee A. L. Aminopropyl-functionalized mesoporous silicas as CO_2_ adsorbents. Fuel Process Technol. 86, 1435–1448 (2005).

[b31] Amornvadee Veawab P. T. & Amit Chakma. Corrosion Behavior of Carbon Steel in the CO_2_ Absorption Process Using Aqueous Amine Solutions. Ind. Eng. Chem. Res. 38, 3917–3924 (1999).

[b32] Xiao D., Yuan D., He H. & Lu J. Microwave-assisted one-step green synthesis of amino-functionalized fluorescent carbon nitride dots from chitosan. Luminescence. 28, 612–615 (2013). 23526712 10.1002/bio.2486

[b33] Wu Q., Li W., Tan J., Wu Y. & Liu S. Hydrothermal carbonization of carboxymethylcellulose: One-pot preparation of conductive carbon microspheres and water-soluble fluorescent carbon nanodots. Chem. Eng. J. 266, 112–120 (2015).

[b34] Li X., Rui M., Song J., Shen Z. & Zeng H. Carbon and Graphene Quantum Dots for Optoelectronic and Energy Devices: A Review. Adv. Funct. Mater. 25, 4929–4947 (2015).

[b35] Luo P. G. *et al.* Carbon-based quantum dots for fluorescence imaging of cells and tissues. RSC Adv. 4, 10791–10807 (2014).

[b36] Wang C. *et al.* A hydrothermal route to water-stable luminescent carbon dots as nanosensors for pH and temperature. Carbon. 82, 87–95 (2015).

[b37] Hola K. *et al.* Photoluminescence effects of graphitic core size and surface functional groups in carbon dots: COO− induced red-shift emission. Carbon. 70, 279–286 (2014).

[b38] Pan D. *et al.* Observation of pH-, solvent-, spin-, and excitation-dependent blue photoluminescence from carbon nanoparticles. Chem Commun (Camb). 46, 3681–3683 (2010). 20396809 10.1039/c000114g

[b39] Wang F. *et al.* Graphene quantum dots as a fluorescent sensing platform for highly efficient detection of copper(II) ions. Sensors Actua B-Chem. 190, 516–522 (2014).

[b40] Zhu S. *et al.* Surface Chemistry Routes to Modulate the Photoluminescence of Graphene Quantum Dots: From Fluorescence Mechanism to Up-Conversion Bioimaging Applications. Adv. Funct. Mater. 22, 4732–4740 (2012).

[b41] Peng J. *et al.* Graphene quantum dots derived from carbon fibers. Nano Lett. 12, 844–849 (2012). 22216895 10.1021/nl2038979

[b42] Liu H., Ye T. & Mao C. Fluorescent carbon nanoparticles derived from candle soot. Angew Chem Int Ed Engl. 46, 6473–6475 (2007). 17645271 10.1002/anie.200701271

[b43] Ling Y. *et al.* Bright Light-Emitting Diodes Based on Organometal Halide Perovskite Nanoplatelets. Adv Mater. 28, 305–311 (2016). 26572239 10.1002/adma.201503954

[b44] Zhang X. *et al.* Carbon-dots derived from nanodiamond: photoluminescence tunable nanoparticles for cell imaging. J. Colloid Interface Sci. 397, 39–44 (2013). 23484769 10.1016/j.jcis.2013.01.063

[b45] Ray Chowdhuri A., Tripathy S., Haldar C., Roy S. & Sahu S. K. Single step synthesis of carbon dot embedded chitosan nanoparticles for cell imaging and hydrophobic drug delivery. J. Mater. Chem. B. 3, 9122–9131 (2015). 10.1039/c5tb01831e 32263126

[b46] Huang C.-L. *et al.* Application of paramagnetic graphene quantum dots as a platform for simultaneous dual-modality bioimaging and tumor-targeted drug delivery. J. Mater. Chem. B. 3, 651–664 (2015). 10.1039/c4tb01650e 32262348

[b47] Tang M., Huang Y., Wang Y. & Fu L. An ytterbium complex with unique luminescence properties: detecting the temperature based on a luminescence spectrum without the interference of oxygen. Dalton Trans. 44, 7449–7457 (2015). 25802201 10.1039/c5dt00611b

[b48] Dorneanu P. P. *et al.* Solvent effects on the photophysical properties of poly[1,4-dihydroxyanthraquinoneimine-1,3-bis(phenylene-ester-methylene)tetramethyldisiloxane]. Spectrochim. Acta, Part A. 134, 218–224 (2015). 10.1016/j.saa.2014.06.104 25014644

[b49] Chen B. *et al.* Fluorescent probe for highly selective and sensitive detection of hydrogen sulfide in living cells and cardiac tissues. Analyst. 138, 946–951 (2013). 23243655 10.1039/c2an36113b

[b50] Li F. *et al.* Mg/N double doping strategy to fabricate extremely high luminescent carbon dots for bioimaging. RSC Adv. 4, 3201–3205 (2014).

